# Botulism in the Brazilian Amazon: a life-threatening disease in a neglected population

**DOI:** 10.1055/s-0042-1758651

**Published:** 2022-12-29

**Authors:** Louise Makarem Oliveira, Daniel Buzaglo Gonçalves, Lucas de Cristo Rojas Cabral, Marília Rosa Abtibol Bernardino, Pablo Vinícius Silveira Feitoza

**Affiliations:** 1 Universidade Federal do Amazonas, Faculdade de Medicina, Manaus AM, Brazil.; 2 Fundação de Medicina Tropical Dr. Heitor Vieira Dourado, Manaus AM, Brazil.

**Keywords:** Botulism, Botulinum Toxins, Neglected Diseases, Botulismo, Toxinas Botulínicas, Doenças Negligenciadas

## Abstract

**Background** Botulism is a rare and potentially fatal neuroparalytic syndrome caused by the gram-positive anaerobe spore-forming bacterium *Clostridium botulinum* . The microorganism produces a neurotoxin that inhibits the presynaptic release of acetylcholine at the neuromuscular junction, clinically leading to a myasthenic syndrome.

**Objective** To describe the recent outbreak of botulism cases and its demographic, clinical, and laboratory characteristics.

**Methods** We report 4 patients with botulism in the recent outbreak occurred between 2017 and 2019 in the state of Amazon.

**Results** Out of four patients with botulism, three contracted it from eating contaminated food and one had wound botulism. We emphasize the excellent clinical outcome of the different disease presentations in our case series.

**Conclusion** The temporal proximity of these reports may suggest a new rise in the number of cases in the upcoming years. A possible hypothesis is that the rarity of the disease decreased the awareness regarding the primary prevention or even a diagnosis by an untrained physician.

## Resumo

**Antecedentes** O botulismo é uma síndrome neuroparalítica rara e potencialmente fatal causada pela bactéria anaeróbia gram-positiva formadora de esporos *Clostridium botulinum.* O microrganismo produz uma neurotoxina que inibe a liberação pré-sináptica de acetilcolina na junção neuromuscular, levando clinicamente a uma síndrome miastênica.

**Objetivo** Descrever o recente surto de casos de botulismo e suas características demográficas, clínicas e laboratoriais.

**Métodos** Relatamos quatro pacientes com botulismo em surto recente ocorrido entre 2017 e 2019 no estado do Amazonas.

**Resultados** Dos quatro pacientes com botulismo, três o contraíram devido ao consumo de alimento contaminado e um por ferimento. Enfatizamos o excelente resultado clínico das diferentes apresentações da doença em nossa série de casos.

**Conclusão** A proximidade temporal dessas notificações pode sugerir um novo aumento no número de casos nos próximos anos. Uma possível hipótese é que a raridade da doença diminuiu a conscientização quanto à prevenção primária ou mesmo ao diagnóstico por médico destreinado.

## INTRODUCTION

Botulism is a rare and potentially fatal neuroparalytic syndrome caused by the gram-positive anaerobe spore-forming bacterium *Clostridium botulinum* . 1 The microorganism produces a neurotoxin that inhibits the presynaptic release of acetylcholine at the neuromuscular junction, clinically leading to a myasthenic syndrome. 2 Botulinum neurotoxins (BoNTs) are produced by a variety of clostridial species. Among more than 200 Clostridium species, 15 produce very potent toxins. Besides *C. botulinum* , we can mention *C. argentinense* , *C. baratii* , and *C. butyricum* as producing organisms. These pathogens are mainly environmental bacteria from soil, sediments, and, occasionally, the intestinal content of man and animals. 3 4 Accidental or intentional exposure to botulinum toxin has led to outbreaks of acute flaccid paralysis worldwide. For example, in 2007, the US Center for Disease Control and Prevention (CDC) reported an outbreak of foodborne botulism associated with a canned hot dog chili sauce. 5 Date et al. also described cases caused by consuming commercial foods and food from food trucks and homemade canned vegetables (green beans, mixed green beans and carrots, and asparagus), which caused botulism outbreaks from 1999 to 2008. 6 In addition, 160 foodborne botulism outbreaks were reported in the United States between 1990 and 2000, reaffirming its epidemiological importance, especially in the pediatric population. 7 Due to the possibility of unfavorable outcomes, the disease is considered a public health issue in Brazil by the Ministry of Health. 8 According to the DATASUS database, 107 cases of botulism were notified between 1999 and 2019 to the health public surveillance system. 9 Between 2018 and 2019, 16 of them were confirmed. Three of those were in Amazonas: 2 reported in 2018, and 1 in 2019. In this article, we present four cases of botulism with an unusually good clinical outcome.

## METHODS

We performed a retrospective study at the Tropical Medicine Foundation Dr. Heitor Vieira Dourado (FMT/HVD), Amazonas, Brazil. We extracted clinical and laboratory data from selected medical records between 2017 and 2019. We searched for the term *botulism* in summarized patient data.

Among the collected data, we characterized the age at onset, gender, city of origin, type of diagnosed botulism, incubation period (in days), symptoms duration, diagnosis criteria, treatment, and electroneuromyography findings. This research was approved by the FMT/HVD ethical committee (CAAE 50719121.8.0000.0005).

## RESULTS

### Patient descriptions

#### *Patient A*

**Figure 1:**
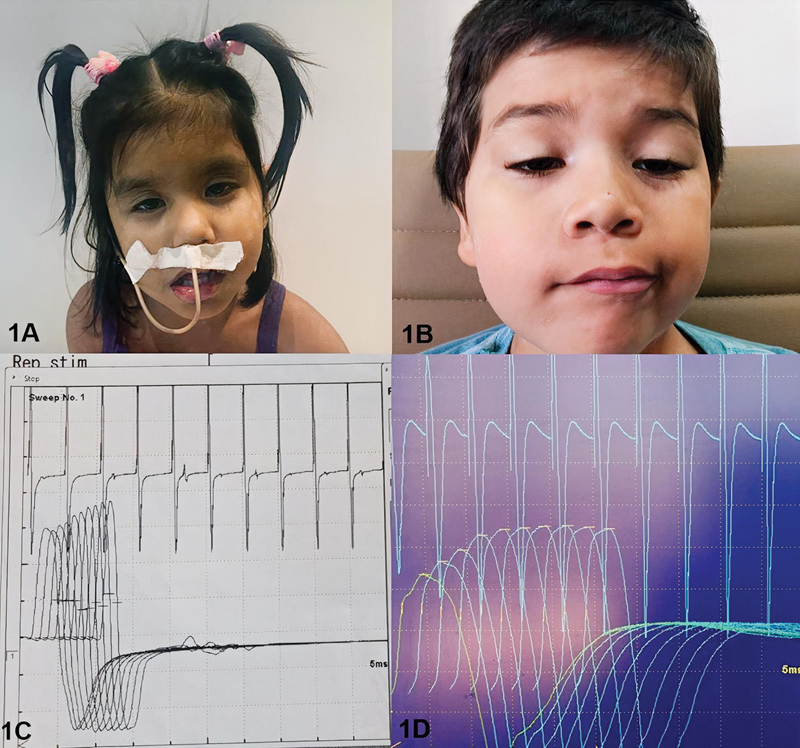
Myasthenic face in a 4-year-old (patient A) female patient with wound botulism (A) and in a 5-year-old (patient B) male with foodborne botulism (B). Electroneuromyography showing potentiation with rapid repetitive stimulation denoting the involvement of the neuromuscular presynaptic junction in patients A and B, respectively (C, D).

A 4-year-old female patient, previously healthy, had an episode of fever and diarrhea. On the next day, she started to present bilateral crural muscle weakness that ascended to her superior limbs. The patient also showed signs of dysphagia and difficulty speaking. Three days after the first symptoms, her family took her to the local hospital in Itacoatiara (countryside of Amazonas). Her previous health history was irrelevant apart from a rusty razor injury 15 days before her weakness onset. The patient was referred to our service after the cause of the clinical presentation couldn't be clarified. On her neurological examination, she was lethargic, globally hypotonic, although preserving the deep tendon reflexes. Interestingly, she had fluctuating bilateral ptosis. Her weakness worsened at the ICU, and due to severe dysphagia, nasogastric intubation was performed ( Figure 1A ). She was misdiagnosed with tetanus and managed as such. After 2 weeks of hospital admission, our team was contacted, and a clinic hypothesis of wound botulism was made. A repetitive examination suggested a presynaptic pattern. Nerve conduction studies corroborated our hypothesis by showing normal sensory nerve action potentials, decreased amplitude of compound muscle action potentials. A presynaptic pattern was found on repetitive stimulation with an amplitude increase of 51% on abductor pollicis brevis muscle at 20 Hz ( Figure 1C ). Apart from cleaning the injury region, no other specific treatment was available due to the diagnostic delay. However, the patient underwent thorough supportive care, and spontaneously improved 1 month after weakness onset. At her last visit, 2 months after disease onset and hospital admission, she had been clinically normal.

#### *Patient B*

A 5-years-old male patient, previously healthy, reported a 6-day history of progressive lower limb pain. On the third day of evolution, progressive gait difficulty was reported by the patient associated also with blurred vision and ptosis. His neurological examination revealed a “myasthenic facies,” with bilateral palpebral ptosis, dysmetria, proximal muscular weakness in the four limbs, ataxic gait, and global hyporeflexia ( Figure 1B ). Family history was noncontributory, including no weakness, neuromuscular disease, or neurodegenerative disorders. Reviewing the clinical history, his mother reported consumption of canned vegetables 4 days before the onset of the symptoms, as well as abdominal pain without diarrhea or vomiting. A diagnostic hypothesis of foodborne botulism was raised, and serum biological samples were performed. The samples sent to Instituto Adolfo Lutz, in São Paulo, later verified the presence of botulinum toxin. His nerve conduction studies confirmed our clinical findings. A presynaptic pattern was found, with an amplitude increase of 89.4% on trapezius muscle at 20 Hz Figure 1D ). On the 8 ^th^ day of evolution, he received an infusion of botulinum antitoxin, following a gradual improvement in the clinical status. The patient was discharged 1 week after the antitoxin infusion, and at the clinical review after 1 month, he presented a global improvement of his muscle strength, mild ptosis, and was able to walk without support.

#### *Patient C*

**Figure 2:**
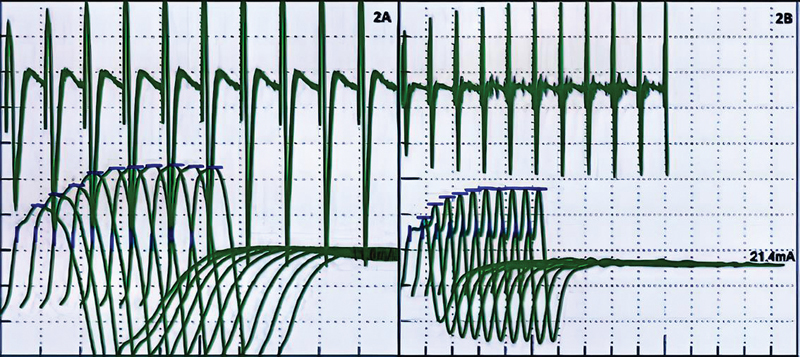
Increment with high-frequency repetitive nerve stimulation (HFRNS) denoting the involvement of the presynaptic neuromuscular junction in the third and fourth patients, respectively.

A 4-year-old indigenous boy from the Tukano ethnic group in the city of São Gabriel da Cachoeira (countryside of the state of Amazonas) presented with abdominal pain and diarrhea after canned food consumption. A week later, he started to present inferior limb paresis evolving to inability to walk or stand. The weakness progressed to his superior limbs. One week after the weakness onset, his parents went to the local hospital, from which they had him transferred to the reference hospital at Manaus. His neurological examination revealed muscular weakness in the four limbs, fatigue, difficulty sitting unsupported, ataxic gait, and his deep tendon reflexes were normal. From his epidemiological history, we evidenced his exposure to contaminated food from canned sardines that were served at his local school. During the disease, there was a substantial improvement in muscle weakness, with no bulbous symptoms, such as dysphonia, dysphagia, or dyspnea. The favorable progression was due to the early use of botulinum antitoxin. His nerve conduction studies showed normal sensory nerve action potentials and compound muscle action potentials. Repetitive stimulation displayed a presynaptic pattern with an amplitude increase of 145.2% on the trapezius muscle at 20 Hz ( Figure 2A ).

#### *Patient D*

The fourth patient was a 1 and a half year-old girl from Novo Airão (countryside of Amazonas). She presented 15 days earlier with facial muscle weakness, progressing in the past week with superior limbs paresis and, later, also presented with inferior limbs weakness. The patient's mother also mentioned dysphagia and walking disability. On her neurological examination, we observed appendicular and facial muscle weakness. Dysphagia and ataxic gait. Additionally, the child—who had already acquired the ability to walk without support—presented with a clear falling tendency. Exposure of the child and other family members who lived in the same house (father, mother, older brother, and maternal grandmother) to contaminated honey was characterized, although only the girl developed the disease. Her nerve conduction studies showed normal sensory nerve action potentials, decreased amplitude of compound muscle action potentials. Repetitive stimulation exposed a presynaptic pattern with an amplitude increase of 372.9% on muscle abductor digiti minimi at 20 Hz ( Figure 2B ). The patient continued to make slow but steady clinical improvement, but gait remains ataxic after hospital discharge.

Subsequent case details for all patients are summarized in Table 1 . Electromyographic findings can be found in Table 2 . All patient symptoms were compared to Clinical Guidelines for Diagnosis and Treatment of Botulism of the CDC in Table 3 . 10

**Table 1:** Demographic and clinical characteristics of the case series

Case	A	B	C	D
Age	4	5	4	18*
Gender	Female	Male	Male	Female
City of origin (state)	Itacoatiara (AM)	Manaus (AM)	São Gabriel da Cachoeira (AM)	Novo Airão (AM)
Type of diagnosed botulism	Wound botulism	Foodborne botulism	Foodborne botulism	Foodborne botulism
Incubation period in days	15	–	9	7
Symptoms duration	14	–	10	21
Diagnosis criteria	Presumptive and electromyographic diagnosis	Presence of botulinum toxin in the patient's serum	Presumptive and electromyographic diagnosis	Presumptive and electromyographic diagnosis
Treatment	Supportive care	Botulinum antitoxin	Botulinum antitoxin	Supportive care

Notes: *, age in months.

Symptoms duration was considered from symptoms onset until hospital discharge.

**Table 2:** Electroneuromyographic findings

Patients	A	B	C	D
SNAP	Normal	Normal	Normal	Normal
Median CMAP				
• Amplitude • Conduction velocity	7.6 mV 51.1 m/s	2.8 mV 50.9 m/s	5.8 mV 58.2 m/s	4.7 mV 64.3 m/s
Peroneal CMAP				
• Amplitude • Conduction velocity	- -	4.3 mV 45.2 m/s	3.4 mV 47.5 m/s	1.2 mV 51.1 m/s
HF-RNS	Amplitude increase of 51.0% on APB muscle at 20 Hz	Amplitude increase of 89.4% on trapezius muscle at 20 Hz	Amplitude increase of 145.2% on trapezius muscle at 20 Hz	Amplitude increase of 372.9% on ADM muscle at 20 Hz

**Abbreviations** : ADM, abdutor digiti minimi; APB, abductor pollicis brevis; CMAP, compound muscle action potential; HF-RNS, high frequency repetitive nerve stimulation; SNAP, sensory nerve action potential.

**Table 3:** Patients' signs and symptoms compared to the most common presentations according to the Clinical Guidelines for Diagnosis and Treatment of Botulism of the Centers for Disease Control and Prevention. 5

	Case			
Most common signs and symptoms	A	B	C	D
Dysphagia	X			X
Blurred vision		X		X
Slurred speech	X			
Difficulty speaking	X	X		
Hoarse voice	X			
Gastrointestinal symptoms	X		X	
Dry mouth				
Shortness of breath	X		X	X
Diplopia				
Descending paralysis				X
Ptosis	X	X		X
Ophthalmoplegia				

## DISCUSSION

Botulism is a rare and potentially lethal condition that may result from different sources: foodborne, wound, infant botulism, adult intestinal toxemia, inhalation, and iatrogenic toxin injection. 11 All of them usually produce the same distinct clinical syndrome of symmetrical cranial nerve palsies followed by dysautonomia and rostrocaudal symmetric flaccid paresis of voluntary muscles, which may progress to respiratory compromise and death. 12 Because diagnostic testing has a low negative predictive value, preemptive therapy is recommended at clinical suspicion. Another justification for quick disease management is that the specific antitoxin can only attack the circulating toxin. Hence, if the patient presents with advanced manifestations such as respiratory failure, the antibodies benefit will likely be very modest compared with the supportive treatment alone. Occasionally, incomplete signs may occur, including isolated diplegia of the cranial nerves sparing extraocular muscles. 13 Variation in clinical presentation has been one of the main reasons for the ongoing challenge that constitutes the diagnosis of botulism. Therefore, isolated cases can be extremely difficult to be considered in the early diagnosis of isolated cases. Instead, only in outbreaks physicians tend to diagnose earlier and establish factors determining causality, especially in cases related to foodborne botulism. 10

Foodborne is the most common form of botulism, and it results from toxin-containing food ingestion. This is usually related to home-canned vegetables. There are numerous reports of foodborne botulism outbreaks, such as the Ohio and Washington State outbreak in 2008 and 2009, that resulted from the ingestion of inadequate vegetable home preparation. 6 Wound botulism is an even rarer disease. Most of the previous reports of such conditions are directly related to exposure to contaminated heroin with the spores of *C. botulinum* . 14 In a recently published case, multiple cutaneous abscesses were present, the suspicion established through a single fiber examination. 15

The neurophysiological evaluation for diagnostic confirmation of presynaptic dysfunction of the neuromuscular junction is a fundamental basis for the diagnosis of botulism, being especially useful in infantile forms. Pathological CMAP postexercise facilitation and incremental response to high-frequency repetitive nerve stimulation (HFRNS) are the most indicative neurophysiologic findings of presynaptic neuromuscular junction (NMJ) dysfunction. One study showed that they were found alone or together in all patients in the acute phase, most patients in the early postacute phase, and a low percentage of patients in the late postacute phase. 16

Botulism prognosis fundamentally depends on early specific therapy and intensive care support. 17 With early recognition, timely initiation of treatment, and meticulous supportive care, patients with botulism typically go on to make a full recovery. 18 We highlight the excellent prognosis of the different disease presentations in our case series. Nonetheless, we emphasize that in one of them, this happened despite an improper initial diagnosis, which hindered antitoxin implementation.

We also call attention to the fact that the Amazon, a Brazilian state, is a large territory with heterogeneously disposed health services, leading to a typical delay in proper medical evaluation. This delay can be extremely deleterious when it comes to botulism. Furthermore, the indigenous populations are at risk for botulism intoxication due to the recent habit of canned alimentation—a mark of the acculturation suffered by many local communities.

Finally, we want to raise the question regarding the immunological maturity and exposure density role in botulism infection. After all, even though families and communities were exposed to contaminated edibles in three of our cases, only the children reported symptoms. On a different note, the good condition prognosis was solely observed in the same group.

The temporal proximity of these reports may suggest a new rise in the number of cases in the upcoming years. A possible hypothesis is that the rarity of the disease decreased the awareness regarding the primary prevention or even a diagnosis by an untrained physician.

In conclusion, botulism is a rare condition that may lead to death if not recognized and treated adequately. Understanding its different forms and the relevance of a high clinical suspicion is a crucial step towards a successful evolution.
